# Undeca­carbon­yl[(4-methyl­sulfanylphen­yl)di­phenyl­phosphane]triruthenium(0): crystal structure and Hirshfeld surface analysis

**DOI:** 10.1107/S2056989018006989

**Published:** 2018-05-15

**Authors:** Omar bin Shawkataly, Hafiz Malik Hussien Abdelnasir, Siti Syaida Sirat, Mukesh M. Jotani, Edward R. T. Tiekink

**Affiliations:** a Chemical Sciences Programme, School of Distance Education, Universiti Sains Malaysia, 11800 USM, Penang, Malaysia; b Department of Chemistry, Alzaiem Alazhari University, 1933, Khartoum, Sudan; c Department of Physics, Bhavan’s Sheth R. A. College of Science, Ahmedabad, Gujarat 380001, India; d Research Centre for Crystalline Materials, School of Science and Technology, Sunway University, 47500 Bandar Sunway, Selangor Darul Ehsan, Malaysia

**Keywords:** crystal structure, ruthenium, cluster, carbon­yl, Hirshfeld surface analysis

## Abstract

The title cluster compound, [Ru_3_(C_19_H_17_PS)(CO)_11_], comprises a triangle of Ru^0^ atoms, two of which are bonded to four carbonyl ligands. The third metal atom is bound to three carbonyl ligands and the phosphane-P atom of a dissymmetric phosphane ligand, PPh_2_(C_6_H_4_SMe-4); no Ru⋯S inter­actions are observed. The phosphane occupies an equatorial position and its proximity to an Ru—Ru edge results in the elongation of this bond with respect to the others [2.8933 (2) Å *cf*. 2.8575 (2) and 2.8594 (3) Å]. In the crystal, phenyl-C—H⋯O(carbon­yl) and carbonyl-O⋯O(carbon­yl) [2.817 (2) Å] inter­actions combine to form a supra­molecular chain propagating along [111]; the chains pack without directional inter­actions between them. The carbonyl-O⋯O(carbon­yl) and other weak contacts have an influence upon the Hirshfeld surfaces with O⋯H contacts making the greatest contribution, *i.e*. 37.4% *cf*. 15.8% for O⋯O and 15.6% for H⋯H contacts.

## Abstract 2

In Ru_3_(CO)_11_PPh_2_(C_6_H_4_SMe-4), the phosphane ligand occupies an equatorial position. In the crystal, phenyl-C—H⋯O(carbon­yl) and carbonyl-O⋯O(carbon­yl) inter­actions general a [111] supra­molecular chain.

## Chemical context   

Tertiary phosphanes (P*R*_3_) have played a major role in the formation and subsequent chemistry of metal carbonyl clusters, often relating to the promising catalytic activity of the products (Bruce *et al.*, 2005 ▸; Shawkataly *et al.*, 2013 ▸; Park *et al.*, 2016 ▸). In general, the thermal reaction of Ru_3_(CO)_12_ with P*R*_3_ leads to Ru_3_(CO)_12 – *n*_(P*R*_3_)_*n*_, *n* = 1–4, cluster compounds (Bruce *et al.*, 1988 ▸, 1989 ▸). The steric and electronic effects of P*R*_3_ often results in the lengthening of Ru—Ru bonds in the Ru_3_ triangle as compared with the parent compound, Ru_3_(CO)_12_, thereby making the cluser more reactive (Bruce *et al.*, 1989 ▸). The PPh_2_C_6_H_4_SMe ligand is of inter­est because it contains two different potential donor groups, *i.e*. P and S, which can result in variable substitution patterns. For example, in the Cu_22_Se_6_(SePh)_10_[PPh_2_(C_6_H_4_SMe)]_8_ cluster, only the P atom of the PPh_2_C_6_H_4_SMe ligand is coordinated to the metal centre while the thio­methyl group remains uncoordinated (Fuhr *et al.*, 2002 ▸). However, the thio­methyl group can further react with other metal atoms to provide opportunities in surface chemistry (Fuhr *et al.*, 2002 ▸). The known crystal structures of triruthenium clusters with the PPh_2_(C_6_H_4_SMe) ligand are surprisingly few in number (Shawkataly *et al.*, 2011*a* ▸,*b* ▸). Herein, the crystal and mol­ecular structures of the title compound, Ru_3_(CO)_11_PPh_2_(C_6_H_4_SMe-4) (I), are described as well as an analysis of the calculated Hirshfeld surface.
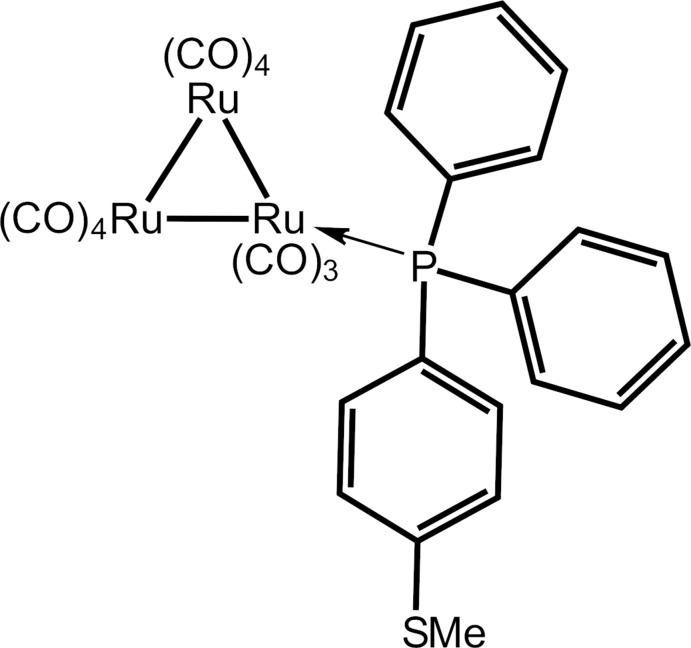


## Structural commentary   

**Figure 1:**
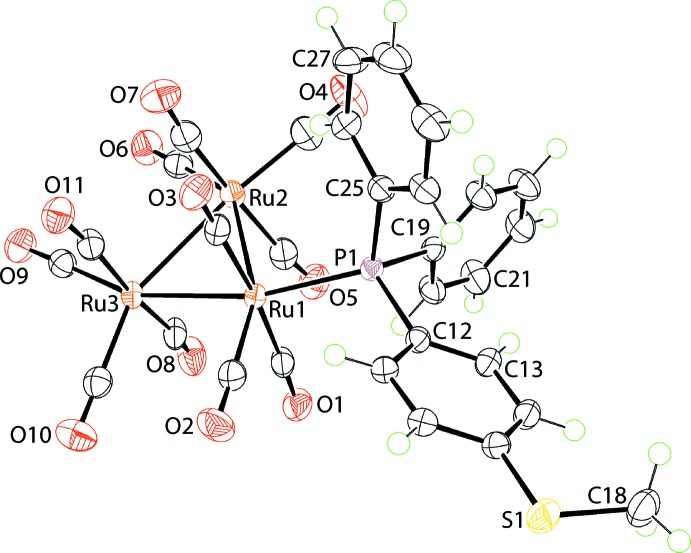
The mol­ecular structure of (I) showing the atom-labelling scheme and displacement ellipsoids at the 70% probability level.

The mol­ecular structure of Ru_3_(CO)_11_PPh_2_(C_6_H_4_SMe-4), (I), is shown in Fig. 1 ▸. The mol­ecule comprises an Ru_3_ triangle with one Ru centre being bound, equatorially, by the phosphane ligand. The Ru—Ru bond lengths in the Ru_3_ triangle are not equivalent with the Ru1—Ru2 bond of 2.8933 (2) Å being longer than the Ru1—Ru3 and Ru2—Ru3 bonds of 2.8575 (2) and 2.8594 (3) Å, respectively. This disparity probably reflects the steric hindrance exerted by the phosphane ligand which occupies the region in the vicinity of the Ru1—Ru2 bond. Some general trends in the geometric parameters involving the carbonyl ligands may be discerned, the relatively high errors in some of the parameters notwithstanding. Thus, the Ru—C bond distances involving carbonyl groups lying in the plane of the Ru_3_ ring are generally shorter than those occupying positions perpendicular to the plane, with the respective ranges in Ru—C bond lengths being 1.897 (3)–1.930 (3) Å and 1.937 (2)–1.953 (3) Å. While the Ru—C≡O angles are all close to linear, two distinctive ranges in angles are evident. The Ru—C≡O angles involving carbonyl groups lying in the plane of the Ru_3_ ring lie in the range 177.3 (2)–178.7 (2)° while the range for the perpendicularly orientated carbonyl groups is 172.1 (2)–174.6 (2)°. The trend for longer Ru—C distances and greater deviations from linearity of the Ru—C≡O angles for the axial carbonyl ligands, which occupy positions *trans* to other carbonyl ligands, is consistent with some semi-bridging character for these carbonyl ligands. Thus, the closest intra­molecular Ru⋯C(carbon­yl) contact of 3.233 (3) Å is formed by the C8-carbonyl ligand which exhibits the maximum deviation from linearity, *i.e*. 172.1 (2)°.

## Supra­molecular features   

**Figure 2:**
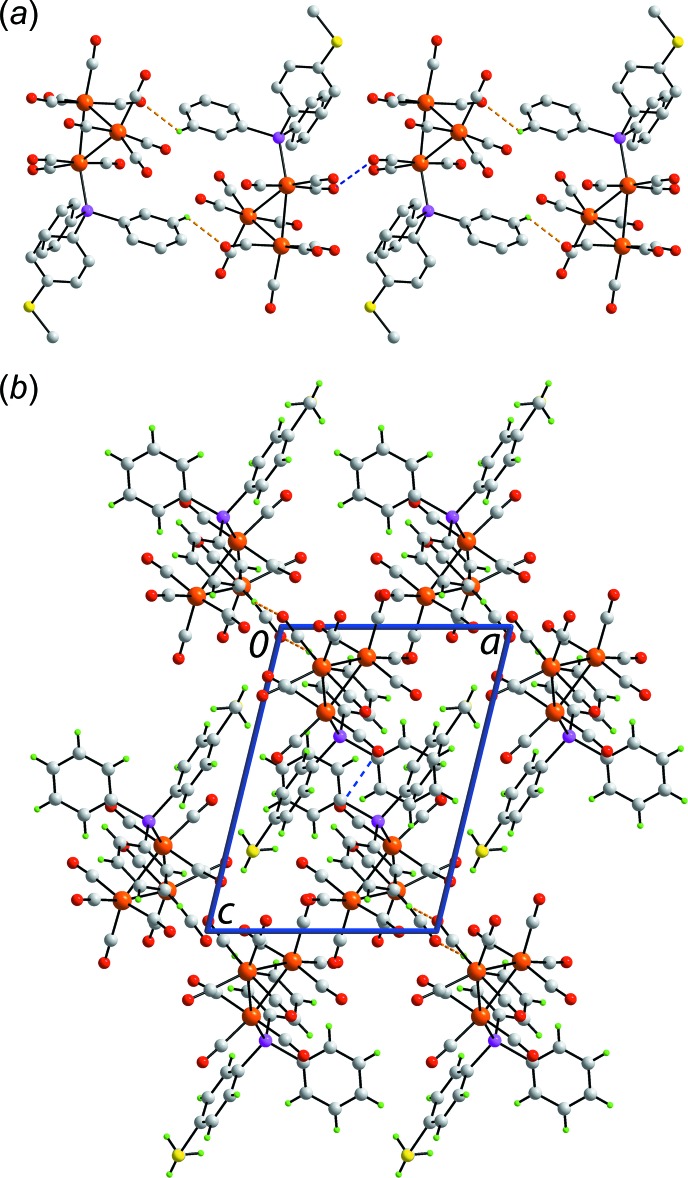
Mol­ecular packing in (I): (*a*) The supra­molecular chain sustained by C—H⋯O and O⋯O inter­actions and (*b*) a view of the unit-cell contents shown in projection down the *b* axis. The C—H⋯O and O⋯O inter­actions are shown as orange and blue dashed lines, respectively.

The mol­ecular packing of (I) features phenyl-C—H⋯O(carbon­yl) inter­actions occurring about a centre of inversion and leading to centrosymmetric dimers, Table 1 ▸. Connections between the dimers leading to a supra­molecular chain along [111] are of the type carbonyl-O⋯O(carbon­yl), Fig. 2 ▸ *a*. The O3⋯O3^i^ separation is 2.817 (2) Å, a distance less than the sum of the van der Waals radii of oxygen, *i.e*. 3.04 Å (Bondi, 1964 ▸); symmetry operation (*i*): 1 − *x*, 1 − *y*, 1 − *z*. Such inter­molecular O⋯O inter­actions are examples of homoatomic chalcogen bonding which are rarest for the smaller oxygen atoms (Gleiter *et al.*, 2018 ▸). The chains pack without directional inter­actions between them according to the criteria assumed in *PLATON* (Spek, 2009 ▸). A view of the unit-cell contents is shown in Fig. 2 ▸ *b*.

## Analysis of the Hirshfeld surface   

**Figure 3:**
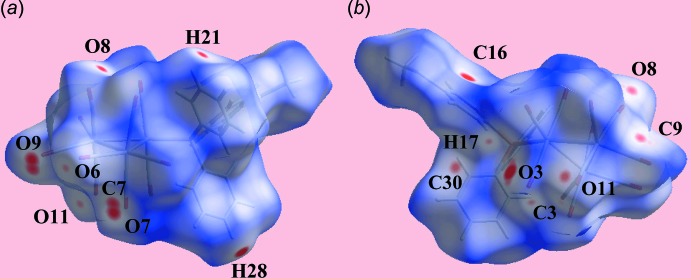
Two views of the Hirshfeld surface of (I) mapped over *d*_norm_ in the range −0.106 to +1.524 au.

**Figure 4:**
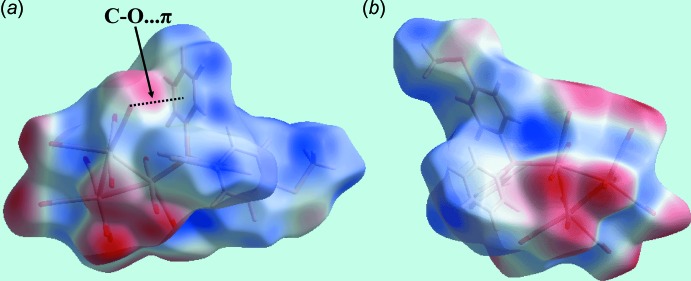
Two views of the Hirshfeld surface of (I) mapped over the electrostatic potential in the range ±0.046 au. The red and blue regions represent negative and positive electrostatic potentials, respectively.

**Figure 5:**
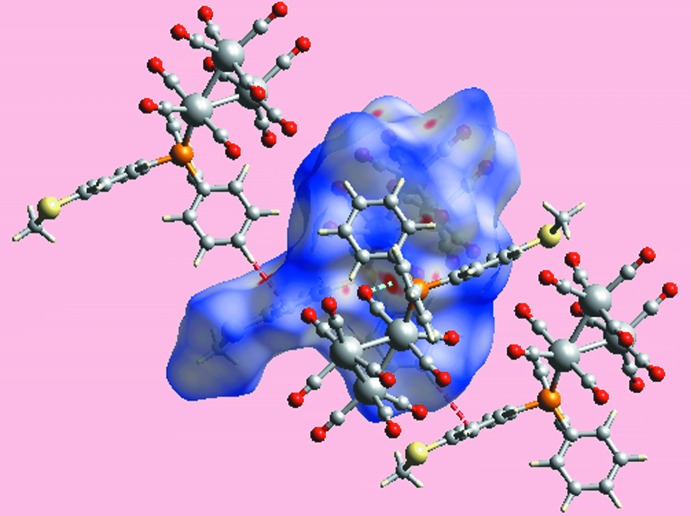
A view of the Hirshfeld surface of (I) mapped over *d*_norm_ in the range −0.090 to +1.204 au highlighting O⋯O and C⋯H/H⋯C contacts by sky-blue and red dashed lines, respectively.

The Hirshfeld surface calculations of (I) were performed in accordance with a recent publication on a related ruthenium cluster compound (Shawkataly *et al.*, 2017 ▸). Two views of the Hirshfeld surface mapped over *d*_norm_ are shown in Fig. 3 ▸. A spot near the O8 atom in Fig. 3 ▸ *a*, results from the C21—H⋯O8 inter­action (Table 1 ▸). The presence of a diminutive red spot near the carbonyl-O3 atom in Fig. 3 ▸ *b* reflects the significance of the short O3⋯O3 contact mentioned in *Supra­molecular features*. The intense red spots near the methyl­sulfanyl­benzene-C16 and phenyl-H28 atoms indicate the significance of this short inter­atomic C⋯H/H⋯C contact (Table 2 ▸; calculated in *CrystalExplorer3.1* (Wolff *et al*., 2012 ▸). In addition, inter­actions involving several carbonyl groups results in short O⋯O and C⋯O/O⋯C contacts (Table 2 ▸) and are characterized as faint red spots in Fig. 3 ▸. The Hirshfeld surfaces mapped over the electrostatic potential illustrated in Fig. 4 ▸ also reflect the involvement of different atoms in the inter­molecular inter­actions through the appearance of blue and red regions around the participating atoms, and correspond to positive and negative electrostatic potential, respectively. As highlighted in Fig. 4 ▸ *a*, an intra­molecular carbonyl-C4≡O4⋯*Cg*(C19–C24) contact is evident. Carbon­yl⋯π(arene) inter­actions are known to be important in the structural chemistry of metal carbonyls (Zukerman-Schpector *et al.*, 2011 ▸). Here, the O4⋯*Cg*(C19–C24) separation is 3.850 (3) Å and the angle subtended at the O4 atom is 90.1 (2)°, indicating a side-on (parallel) approach between the residues. The environment about a reference mol­ecule, showing short inter­atomic O⋯O and C⋯H/H⋯C contacts significant in the mol­ecule packing of (I), is illustrated in Fig. 5 ▸.

**Figure 6:**
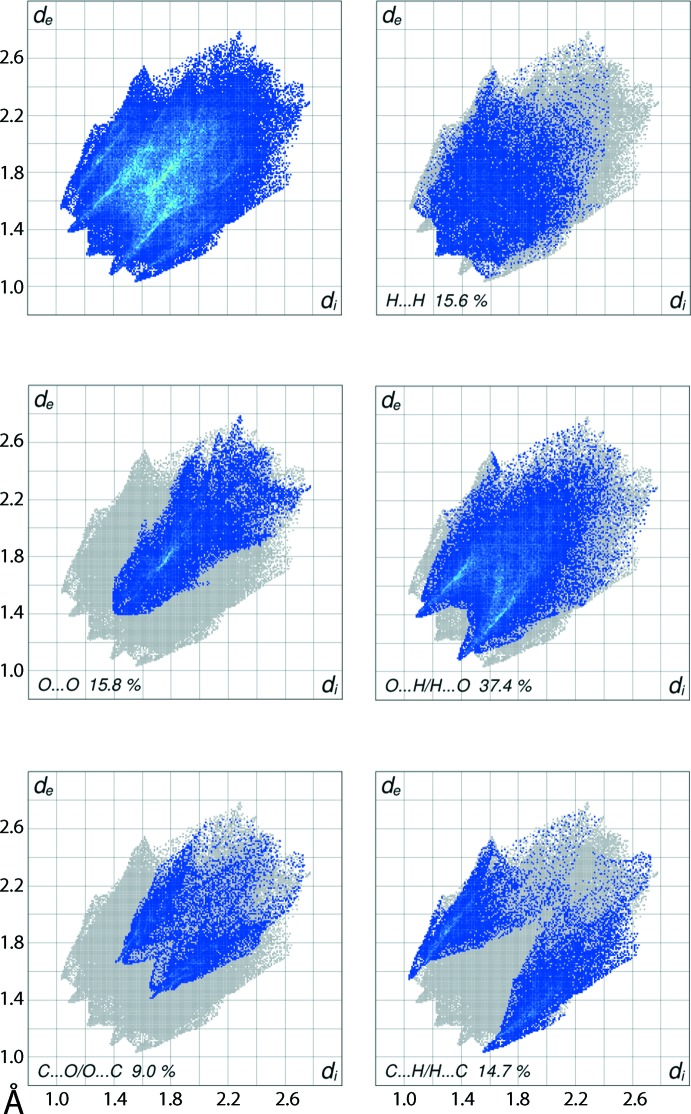
The full two-dimensional fingerprint plot for (I) and those delineated into H⋯H, O⋯H/H⋯O, O⋯O, C⋯H/H⋯C and C⋯O/O⋯C contacts.

The overall two-dimensional fingerprint plot for (I) and those delineated into H⋯H, O⋯H/H⋯O, O⋯O, C⋯H/H⋯C and C⋯O/O⋯C contacts (McKinnon *et al.*, 2007 ▸) are illustrated in Fig. 6 ▸; the percentage contributions from the different inter­atomic contacts to the Hirshfeld surfaces are summarized in Table 3 ▸. In the fingerprint plot delineated into H⋯H contacts, the relatively small, *i.e*. 15.6%, contribution from these contacts to the Hirshfeld surfaces is due to the presence of the carbonyl groups on the Ru-cluster which leads to an increase in the contribution of O⋯H/H⋯O contacts to the Hirshfeld surface, *i.e*. 37.4%. The single tip at *d*_e_ + *d*_i_ ∼2.4 Å in the H⋯H delineated fingerprint plot, which has a broad appearance, arises from a van der Waals contact between the methyl-H18*B* and phenyl-H20 atoms (Table 2 ▸). The two pairs of adjacent peaks at *d*_e_ + *d*_i_ ∼2.5 and 2.6 Å in the fingerprint plot delineated into O⋯H/H⋯O contacts are the result of the inter­atomic C—H⋯O inter­action discussed above (Table 1 ▸) and a short inter­atomic O⋯H/H⋯O contact (Table 2 ▸), respectively. The influence of the significant inter­atomic O3⋯O3 contact (Fig. 5 ▸) and other such short inter­atomic contacts (Table 3 ▸) are viewed as the distribution of points with the rocket-like tip extending from *d*_e_ + *d*_i_ ∼2.8 Å in the plot delineated into O⋯O contacts. In the fingerprint plot delineated into C⋯O/O⋯C contacts, the short inter­atomic contacts between carbonyl-C7 and -O9 atoms appear as the pair of thin tips at *d*_e_ + *d*_i_ ∼3.1 Å superimposed on the parabolic distribution of points characterizing other such short inter­atomic contacts through the points around *d*_e_ = *d*_i_ = 1.6 Å. The other dominant short inter­atomic C⋯H/H⋯C contacts (Table 2 ▸) result in the pair of forceps-like tips at *d*_e_ + *d*_i_ ∼2.6 Å in the respective delineated fingerprint plot. The small contribution from other remaining inter­atomic contacts summarized in Table 3 ▸ have negligible effect on the packing.

## Database survey   

As mentioned in the *Chemical context*, there are two other Ru_3_ clusters in the literature having the same (4-methyl­sulfanylphen­yl)di­phenyl­phosphane ligand as in (I). These are formulated as Ru_3_(CO)_9_PPh_2_(C_6_H_4_SMe-4)(Ph_2_PCH_2_PPh_2_) (II) (Shawkataly *et al.*, 2011*b* ▸) and its arsenic analogue, Ru_3_(CO)_9_PPh_2_(C_6_H_4_SMe-4)(Ph_2_AsCH_2_AsPh_2_) (Shawkataly *et al.*, 2011*a* ▸), in each of which the bidentate ligand bridges the other two ruthenium atoms in the triangle. The structural motif found in (I), *i.e*. with an equatorially substituted phosphane ligand, is consistent with the approximately 35 literature precedents with the general formula Ru_3_(CO)_11_P*RR*′*R*′′ and several examples where the phosphane ligand is bidentate bridging, *i.e*. Ru_3_(CO)_11_P*R*(*R*′)–*R*′′–(*R*′)*R*PRu_3_(CO)_11_ (Groom *et al.*, 2016 ▸). There are no crystallographic examples with perpendicular mono-substitution of phosphane ligands in Ru_3_(CO)_11_P*RR*′*R*′′.

## Synthesis and crystallization   

All reactions were carried out under an inert atmosphere of oxygen-free nitro­gen (OFN) using standard Schlenk techniques. Ru_3_(CO)_12_ was purchased from Aldrich and PPh_2_C_6_H_4_SMe was synthesized as reported previously (Fuhr *et al.*, 2002 ▸). Ru_3_(CO)_11_P(C_6_H_4_SMe-4)Ph_2_ (I) was synthesized by dissolving Ru_3_(CO)_12_ (100 mg, 0.0015 mmol) and PPh_2_(C_6_H_4_SMe) (48 mg, 0.0015 mmol) in tetra­hydro­furan (25 ml). The reaction mixture was treated dropwise with sodium di­phenyl­ketyl solution until the colour of the mixture turned from orange to dark red and then stirred for 30 min. The solvent was evaporated under vacuum and the residue was chromatographed by preparative TLC. Elution with 7:3 *n*-hexa­ne/di­chloro­methane mixture gave four bands and the major orange fraction was characterized as (I) (117 mg, 79.6%). Orange crystals were crystallized from solvent diffusion of di­chloro­methane into a methanol solution of (I). Analysis calculated for C_30_H_17_O_11_PRu_3_S: C, 39.18; H, 1.86%. Found: C, 39.60; H, 1.90%. IR (C_6_H_12_): ν(CO) 2097(*m*), 2059(*w*), 2046(*m*), 2015(*s*), 1989(*w*) cm^−1^. ^1^H NMR (CDCl_3_): δ 7.45–7.23 (*m*, 14H, Ph, C_6_H_4_), 2.48 (*s*, Me). ^13^C NMR (CDCl_3_): δ 204.24 (Ru—CO), 135.19–125.37 (Ph), 14.79 (Me). ^31^P NMR (CDCl_3_): δ 34.28 (*s*).

## Refinement   

Crystal data, data collection and structure refinement details are summarized in Table 4 ▸. The carbon-bound H atoms were placed in calculated positions (C—H = 0.95–0.98 Å) and were included in the refinement in the riding-model approximation, with *U*_iso_(H) set to 1.2–1.5*U*_eq_(C). Owing to poor agreement, four reflections, *i.e*. (1 7 14), (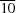 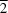 6), (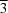 12 12) and (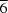 16 10), were omitted from the final cycles of refinement. The maximum and minimum residual electron density peaks of 1.97 and 0.98 e Å^−3^, respectively, were located 0.69 and 0.61 Å from the atoms Ru1 and Ru3, respectively.

## Supplementary Material

Crystal structure: contains datablock(s) I, global. DOI: 10.1107/S2056989018006989/hb7749sup1.cif

Structure factors: contains datablock(s) I. DOI: 10.1107/S2056989018006989/hb7749Isup2.hkl

CCDC reference: 1842044

Additional supporting information:  crystallographic information; 3D view; checkCIF report

## Figures and Tables

**Table 1:** Hydrogen-bond geometry (Å, °)

*D*—H⋯*A*	*D*—H	H⋯*A*	*D*⋯*A*	*D*—H⋯*A*
C21—H21⋯O8^i^	0.95	2.55	3.238 (3)	129

Symmetry code: (i) 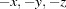.

**Table 2:** Summary of short inter­atomic contacts (Å) in (I)

Contact	Distance	Symmetry operation
O3⋯O3	2.817 (2)	1 − *x*, 1 − *y*, 1 − *z*
O6⋯O11	2.986 (3)	1 − *x*, 1 − *y*, − *z*
C3⋯O3	3.150 (3)	1 − *x*, 1 − *y*, 1 − *z*
C7⋯O9	3.088 (3)	1 − *x*, 1 − *y*, − *z*
C9⋯O8	3.137 (3)	−*x*, 1 − *y*, − *z*
C17⋯O11	3.196 (3)	1 − *x*, 1 − *y*, 1 − *z*
C30⋯O11	3.122 (3)	1 − *x*, 1 − *y*, 1 − *z*
C16⋯H28	2.59	−1 + *x*, *y*, *z*
O3⋯H17	2.54	1 − *x*, 1 − *y*, 1 − *z*
H18*B*⋯H20	2.44	−*x*, −*y*, 1 − *z*

**Table 3:** Percentage contributions of inter­atomic contacts to the Hirshfeld surface for (I)

Contact	Percentage contribution
H⋯H	15.6
O⋯H/H⋯O	37.4
C⋯H/H⋯C	14.7
O⋯O	15.8
C⋯O/O⋯C	9.0
S⋯H/H⋯S	2.6
S⋯O/O⋯S	2.4
C⋯C	1.6
C⋯S/S⋯C	0.9

**Table 4:** Experimental details

Crystal data	
Chemical formula	[Ru_3_(C_19_H_17_PS)(CO)_11_]
*M*_r_	919.67
Crystal system, space group	Triclinic, *P* 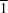
Temperature (K)	100
*a*, *b*, *c* (Å)	9.6922 (1), 12.7459 (2), 13.6030 (2)
α, β, γ (°)	103.301 (1), 102.938 (1), 91.771 (1)
*V* (Å^3^)	1587.83 (4)
*Z*	2
Radiation type	Mo *K*α
μ (mm^−1^)	1.58
Crystal size (mm)	0.32 × 0.30 × 0.14

Data collection	
Diffractometer	Bruker SMART APEXII CCD
Absorption correction	Multi-scan (*SADABS*; Bruker, 2009 ▸)
*T*_min_, *T*_max_	0.448, 0.526
No. of measured, independent and observed [*I* > 2σ(*I*)] reflections	56976, 15652, 11725
*R*_int_	0.042
(sin θ/λ)_max_ (Å^−1^)	0.842

Refinement	
*R*[*F* ^2^ > 2σ(*F* ^2^)], *wR*(*F* ^2^), *S*	0.041, 0.097, 1.03
No. of reflections	15652
No. of parameters	416
H-atom treatment	H-atom parameters constrained
Δρ_max_, Δρ_min_ (e Å^−3^)	1.97, −0.98

Computer programs: *APEX2* and *SAINT* (Bruker, 2009 ▸), *SHELXS* (Sheldrick, 2008 ▸), *SHELXL2014* (Sheldrick, 2015 ▸), *ORTEP-3 for Windows* (Farrugia, 2012 ▸), *DIAMOND* (Brandenburg, 2006 ▸) and *publCIF* (Westrip, 2010 ▸).

## supplementary crystallographic information

### Crystal data

**Table:**

[Ru_3_(C_19_H_17_PS)(CO)_11_]	*Z* = 2
*M**_r_* = 919.67	*F*(000) = 896
Triclinic, *P*1	*D*_x_ = 1.924 Mg m^−^^3^
*a* = 9.6922 (1) Å	Mo *K*α radiation, λ = 0.71073 Å
*b* = 12.7459 (2) Å	Cell parameters from 9414 reflections
*c* = 13.6030 (2) Å	θ = 2.6–36.5°
α = 103.301 (1)°	µ = 1.58 mm^−^^1^
β = 102.938 (1)°	*T* = 100 K
γ = 91.771 (1)°	Block, orange
*V* = 1587.83 (4) Å^3^	0.32 × 0.30 × 0.14 mm

### Data collection

**Table:**

Bruker SMART APEXII CCD diffractometer	11725 reflections with *I* > 2σ(*I*)
Radiation source: fine-focus sealed tube	*R*_int_ = 0.042
φ and ω scans	θ_max_ = 36.8°, θ_min_ = 2.0°
Absorption correction: multi-scan (*SADABS*; Bruker, 2009)	*h* = −16→16
*T*_min_ = 0.448, *T*_max_ = 0.526	*k* = −21→21
56976 measured reflections	*l* = −22→22
15652 independent reflections	

### Refinement

**Table:**

Refinement on *F*^2^	Primary atom site location: structure-invariant direct methods
Least-squares matrix: full	Hydrogen site location: inferred from neighbouring sites
*R*[*F*^2^ > 2σ(*F*^2^)] = 0.041	H-atom parameters constrained
*wR*(*F*^2^) = 0.097	*w* = 1/[σ^2^(*F*_o_^2^) + (0.048*P*)^2^] where *P* = (*F*_o_^2^ + 2*F*_c_^2^)/3
*S* = 1.03	(Δ/σ)_max_ = 0.001
15652 reflections	Δρ_max_ = 1.97 e Å^−^^3^
416 parameters	Δρ_min_ = −0.98 e Å^−^^3^
0 restraints	

### Special details

**Table:**

Geometry. All esds (except the esd in the dihedral angle between two l.s. planes) are estimated using the full covariance matrix. The cell esds are taken into account individually in the estimation of esds in distances, angles and torsion angles; correlations between esds in cell parameters are only used when they are defined by crystal symmetry. An approximate (isotropic) treatment of cell esds is used for estimating esds involving l.s. planes.

### Fractional atomic coordinates and isotropic or equivalent isotropic displacement parameters (Å^2^)

**Table:**

	*x*	*y*	*z*	*U*_iso_*/*U*_eq_	
Ru1	0.28387 (2)	0.33186 (2)	0.28051 (2)	0.01428 (4)	
Ru2	0.40348 (2)	0.29273 (2)	0.09996 (2)	0.01862 (4)	
Ru3	0.21842 (2)	0.46106 (2)	0.13284 (2)	0.01626 (4)	
S1	0.11319 (7)	0.13142 (5)	0.73844 (5)	0.02529 (12)	
P1	0.37139 (6)	0.19229 (4)	0.36008 (4)	0.01376 (10)	
O1	−0.00653 (19)	0.21034 (14)	0.16345 (15)	0.0282 (4)	
O2	0.1200 (2)	0.45096 (15)	0.43099 (15)	0.0308 (4)	
O3	0.55312 (18)	0.47646 (14)	0.40979 (13)	0.0251 (4)	
O4	0.6007 (3)	0.11066 (18)	0.10236 (18)	0.0435 (5)	
O5	0.1625 (2)	0.11127 (15)	−0.00206 (15)	0.0312 (4)	
O6	0.4152 (2)	0.34976 (16)	−0.10467 (15)	0.0309 (4)	
O7	0.6584 (2)	0.45662 (17)	0.22391 (15)	0.0319 (4)	
O8	−0.0055 (2)	0.29918 (15)	−0.03258 (16)	0.0326 (4)	
O9	0.2453 (2)	0.57908 (15)	−0.03402 (14)	0.0288 (4)	
O10	−0.0099 (2)	0.57899 (17)	0.22667 (16)	0.0335 (4)	
O11	0.4558 (2)	0.62755 (14)	0.27933 (14)	0.0273 (4)	
C1	0.1048 (3)	0.25236 (18)	0.20139 (19)	0.0207 (4)	
C2	0.1833 (3)	0.40534 (19)	0.37650 (19)	0.0215 (4)	
C3	0.4559 (3)	0.42297 (18)	0.35766 (18)	0.0195 (4)	
C4	0.5271 (3)	0.1777 (2)	0.1037 (2)	0.0279 (5)	
C5	0.2456 (3)	0.1817 (2)	0.03905 (19)	0.0246 (5)	
C6	0.4106 (3)	0.3274 (2)	−0.0295 (2)	0.0236 (5)	
C7	0.5600 (3)	0.4000 (2)	0.18099 (19)	0.0238 (5)	
C8	0.0801 (3)	0.35306 (19)	0.03187 (19)	0.0228 (5)	
C9	0.2348 (3)	0.53252 (19)	0.02662 (18)	0.0211 (4)	
C10	0.0760 (3)	0.53550 (19)	0.19383 (19)	0.0223 (4)	
C11	0.3718 (3)	0.56093 (19)	0.22852 (19)	0.0215 (4)	
C12	0.2835 (2)	0.16989 (17)	0.45989 (17)	0.0155 (4)	
C13	0.2366 (2)	0.06656 (18)	0.46472 (17)	0.0174 (4)	
H13	0.2421	0.0056	0.4108	0.021*	
C14	0.1818 (2)	0.05201 (18)	0.54780 (18)	0.0189 (4)	
H14	0.1480	−0.0183	0.5491	0.023*	
C15	0.1765 (2)	0.14016 (18)	0.62855 (17)	0.0179 (4)	
C16	0.2236 (2)	0.24400 (18)	0.62442 (17)	0.0176 (4)	
H16	0.2203	0.3048	0.6792	0.021*	
C17	0.2748 (2)	0.25804 (17)	0.54035 (17)	0.0166 (4)	
H17	0.3045	0.3288	0.5376	0.020*	
C18	0.1152 (3)	−0.0110 (2)	0.7356 (2)	0.0276 (5)	
H18A	0.0428	−0.0525	0.6756	0.041*	
H18B	0.0949	−0.0232	0.7997	0.041*	
H18C	0.2090	−0.0346	0.7299	0.041*	
C19	0.3634 (2)	0.05642 (16)	0.27629 (16)	0.0153 (4)	
C20	0.2408 (2)	0.01554 (18)	0.19821 (19)	0.0216 (4)	
H20	0.1635	0.0592	0.1886	0.026*	
C21	0.2302 (3)	−0.08793 (19)	0.1345 (2)	0.0268 (5)	
H21	0.1462	−0.1145	0.0818	0.032*	
C22	0.3416 (3)	−0.15220 (19)	0.1476 (2)	0.0253 (5)	
H22	0.3348	−0.2228	0.1037	0.030*	
C23	0.4638 (3)	−0.1130 (2)	0.2255 (2)	0.0264 (5)	
H23	0.5405	−0.1572	0.2350	0.032*	
C24	0.4747 (2)	−0.00928 (18)	0.28960 (19)	0.0214 (4)	
H24	0.5585	0.0168	0.3427	0.026*	
C25	0.5593 (2)	0.21583 (17)	0.43099 (17)	0.0171 (4)	
C26	0.6609 (3)	0.24650 (19)	0.3821 (2)	0.0224 (4)	
H26	0.6323	0.2535	0.3126	0.027*	
C27	0.8033 (3)	0.2669 (2)	0.4343 (2)	0.0282 (5)	
H27	0.8715	0.2876	0.4003	0.034*	
C28	0.8460 (3)	0.2571 (2)	0.5359 (2)	0.0288 (5)	
H28	0.9431	0.2725	0.5721	0.035*	
C29	0.7475 (3)	0.2249 (2)	0.5841 (2)	0.0266 (5)	
H29	0.7772	0.2172	0.6533	0.032*	
C30	0.6042 (2)	0.20356 (18)	0.53226 (18)	0.0195 (4)	
H30	0.5371	0.1806	0.5660	0.023*	

### Atomic displacement parameters (Å^2^)

**Table:**

	*U*^11^	*U*^22^	*U*^33^	*U*^12^	*U*^13^	*U*^23^
Ru1	0.01599 (8)	0.01239 (7)	0.01479 (7)	0.00070 (5)	0.00376 (6)	0.00392 (6)
Ru2	0.02282 (9)	0.01803 (8)	0.01699 (8)	0.00456 (7)	0.00739 (7)	0.00527 (6)
Ru3	0.01832 (8)	0.01340 (7)	0.01637 (8)	0.00103 (6)	0.00223 (6)	0.00411 (6)
S1	0.0303 (3)	0.0269 (3)	0.0244 (3)	0.0041 (2)	0.0138 (2)	0.0103 (2)
P1	0.0135 (2)	0.0127 (2)	0.0147 (2)	0.00001 (18)	0.00275 (18)	0.00325 (18)
O1	0.0219 (9)	0.0246 (9)	0.0366 (10)	−0.0025 (7)	0.0012 (8)	0.0107 (8)
O2	0.0334 (10)	0.0308 (10)	0.0317 (10)	0.0124 (8)	0.0161 (8)	0.0052 (8)
O3	0.0263 (9)	0.0226 (8)	0.0236 (9)	−0.0045 (7)	0.0038 (7)	0.0031 (7)
O4	0.0515 (14)	0.0407 (12)	0.0419 (12)	0.0280 (11)	0.0132 (11)	0.0125 (10)
O5	0.0386 (11)	0.0241 (9)	0.0308 (10)	−0.0003 (8)	0.0110 (8)	0.0042 (8)
O6	0.0403 (11)	0.0320 (10)	0.0250 (9)	0.0068 (8)	0.0130 (8)	0.0103 (8)
O7	0.0289 (10)	0.0379 (11)	0.0280 (10)	−0.0020 (8)	0.0092 (8)	0.0046 (8)
O8	0.0327 (10)	0.0215 (9)	0.0355 (10)	−0.0014 (7)	−0.0062 (8)	0.0053 (8)
O9	0.0333 (10)	0.0297 (9)	0.0255 (9)	0.0008 (8)	0.0080 (8)	0.0103 (8)
O10	0.0272 (10)	0.0376 (11)	0.0334 (10)	0.0080 (8)	0.0092 (8)	0.0014 (9)
O11	0.0287 (9)	0.0231 (8)	0.0266 (9)	−0.0030 (7)	0.0032 (7)	0.0032 (7)
C1	0.0237 (11)	0.0159 (9)	0.0239 (11)	0.0033 (8)	0.0049 (9)	0.0080 (8)
C2	0.0216 (11)	0.0206 (10)	0.0238 (11)	0.0024 (8)	0.0051 (9)	0.0087 (9)
C3	0.0240 (11)	0.0162 (9)	0.0196 (10)	0.0013 (8)	0.0064 (8)	0.0059 (8)
C4	0.0329 (14)	0.0297 (13)	0.0233 (12)	0.0088 (11)	0.0091 (10)	0.0081 (10)
C5	0.0319 (13)	0.0226 (11)	0.0215 (11)	0.0054 (10)	0.0099 (10)	0.0060 (9)
C6	0.0249 (12)	0.0235 (11)	0.0237 (11)	0.0037 (9)	0.0092 (9)	0.0051 (9)
C7	0.0284 (12)	0.0250 (11)	0.0203 (10)	0.0060 (9)	0.0094 (9)	0.0061 (9)
C8	0.0252 (12)	0.0165 (10)	0.0257 (11)	0.0008 (8)	0.0013 (9)	0.0081 (9)
C9	0.0211 (11)	0.0197 (10)	0.0212 (10)	0.0018 (8)	0.0038 (8)	0.0033 (8)
C10	0.0222 (11)	0.0212 (10)	0.0219 (11)	−0.0004 (9)	0.0024 (9)	0.0054 (9)
C11	0.0227 (11)	0.0185 (10)	0.0227 (11)	0.0024 (8)	0.0035 (9)	0.0057 (8)
C12	0.0141 (9)	0.0159 (9)	0.0166 (9)	0.0008 (7)	0.0019 (7)	0.0061 (7)
C13	0.0177 (10)	0.0168 (9)	0.0175 (9)	−0.0010 (7)	0.0031 (8)	0.0054 (8)
C14	0.0183 (10)	0.0183 (10)	0.0210 (10)	−0.0005 (8)	0.0039 (8)	0.0075 (8)
C15	0.0154 (10)	0.0205 (10)	0.0197 (10)	0.0025 (8)	0.0050 (8)	0.0079 (8)
C16	0.0163 (10)	0.0185 (9)	0.0180 (9)	0.0022 (8)	0.0044 (8)	0.0039 (8)
C17	0.0151 (9)	0.0167 (9)	0.0183 (9)	0.0012 (7)	0.0038 (7)	0.0050 (8)
C18	0.0314 (13)	0.0298 (13)	0.0268 (12)	0.0003 (10)	0.0104 (10)	0.0143 (10)
C19	0.0157 (9)	0.0127 (8)	0.0167 (9)	−0.0003 (7)	0.0041 (7)	0.0023 (7)
C20	0.0174 (10)	0.0169 (10)	0.0276 (11)	0.0012 (8)	−0.0001 (9)	0.0047 (9)
C21	0.0240 (12)	0.0170 (10)	0.0305 (13)	−0.0020 (9)	−0.0058 (10)	0.0002 (9)
C22	0.0298 (13)	0.0149 (10)	0.0262 (12)	0.0010 (9)	0.0040 (10)	−0.0022 (9)
C23	0.0249 (12)	0.0192 (11)	0.0315 (13)	0.0068 (9)	0.0044 (10)	0.0005 (9)
C24	0.0182 (10)	0.0195 (10)	0.0223 (10)	0.0028 (8)	−0.0002 (8)	0.0012 (8)
C25	0.0146 (9)	0.0146 (9)	0.0200 (10)	0.0002 (7)	0.0018 (8)	0.0024 (8)
C26	0.0195 (11)	0.0222 (11)	0.0271 (11)	0.0001 (8)	0.0064 (9)	0.0087 (9)
C27	0.0156 (11)	0.0257 (12)	0.0424 (15)	−0.0003 (9)	0.0072 (10)	0.0063 (11)
C28	0.0152 (11)	0.0236 (11)	0.0397 (15)	0.0009 (9)	−0.0024 (10)	0.0006 (11)
C29	0.0243 (12)	0.0238 (11)	0.0244 (11)	0.0048 (9)	−0.0031 (9)	−0.0003 (9)
C30	0.0174 (10)	0.0177 (9)	0.0203 (10)	0.0032 (8)	0.0014 (8)	0.0015 (8)

### Geometric parameters (Å, º)

**Table:**

Ru1—C2	1.897 (3)	C13—C14	1.397 (3)
Ru1—C1	1.937 (2)	C13—H13	0.9500
Ru1—C3	1.941 (2)	C14—C15	1.390 (3)
Ru1—P1	2.3714 (5)	C14—H14	0.9500
Ru1—Ru3	2.8575 (2)	C15—C16	1.404 (3)
Ru1—Ru2	2.8933 (2)	C16—C17	1.389 (3)
Ru2—C4	1.924 (3)	C16—H16	0.9500
Ru2—C6	1.927 (2)	C17—H17	0.9500
Ru2—C5	1.946 (3)	C18—H18A	0.9800
Ru2—C7	1.953 (3)	C18—H18B	0.9800
Ru2—Ru3	2.8594 (3)	C18—H18C	0.9800
Ru3—C9	1.908 (2)	C19—C24	1.392 (3)
Ru3—C10	1.930 (3)	C19—C20	1.398 (3)
Ru3—C8	1.942 (2)	C20—C21	1.388 (3)
Ru3—C11	1.944 (2)	C20—H20	0.9500
S1—C15	1.763 (2)	C21—C22	1.380 (4)
S1—C18	1.808 (3)	C21—H21	0.9500
P1—C12	1.826 (2)	C22—C23	1.390 (4)
P1—C19	1.830 (2)	C22—H22	0.9500
P1—C25	1.840 (2)	C23—C24	1.393 (3)
O1—C1	1.142 (3)	C23—H23	0.9500
O2—C2	1.136 (3)	C24—H24	0.9500
O3—C3	1.138 (3)	C25—C30	1.395 (3)
O4—C4	1.130 (3)	C25—C26	1.397 (3)
O5—C5	1.136 (3)	C26—C27	1.389 (3)
O6—C6	1.133 (3)	C26—H26	0.9500
O7—C7	1.134 (3)	C27—C28	1.387 (4)
O8—C8	1.136 (3)	C27—H27	0.9500
O9—C9	1.141 (3)	C28—C29	1.375 (4)
O10—C10	1.132 (3)	C28—H28	0.9500
O11—C11	1.139 (3)	C29—C30	1.397 (3)
C12—C17	1.396 (3)	C29—H29	0.9500
C12—C13	1.401 (3)	C30—H30	0.9500
			
C2—Ru1—C1	87.40 (10)	O11—C11—Ru3	172.7 (2)
C2—Ru1—C3	90.22 (10)	C17—C12—C13	118.4 (2)
C1—Ru1—C3	174.97 (9)	C17—C12—P1	118.62 (15)
C2—Ru1—P1	101.13 (7)	C13—C12—P1	122.67 (17)
C1—Ru1—P1	95.84 (6)	C14—C13—C12	120.9 (2)
C3—Ru1—P1	88.97 (7)	C14—C13—H13	119.6
C2—Ru1—Ru3	97.52 (7)	C12—C13—H13	119.6
C1—Ru1—Ru3	83.06 (6)	C15—C14—C13	120.2 (2)
C3—Ru1—Ru3	92.87 (6)	C15—C14—H14	119.9
P1—Ru1—Ru3	161.254 (16)	C13—C14—H14	119.9
C2—Ru1—Ru2	156.94 (7)	C14—C15—C16	119.3 (2)
C1—Ru1—Ru2	92.30 (7)	C14—C15—S1	124.25 (17)
C3—Ru1—Ru2	88.15 (7)	C16—C15—S1	116.43 (17)
P1—Ru1—Ru2	101.829 (15)	C17—C16—C15	120.1 (2)
Ru3—Ru1—Ru2	59.628 (6)	C17—C16—H16	119.9
C4—Ru2—C6	102.37 (11)	C15—C16—H16	119.9
C4—Ru2—C5	87.57 (11)	C16—C17—C12	121.1 (2)
C6—Ru2—C5	95.01 (10)	C16—C17—H17	119.4
C4—Ru2—C7	91.05 (11)	C12—C17—H17	119.5
C6—Ru2—C7	93.55 (10)	S1—C18—H18A	109.5
C5—Ru2—C7	171.43 (10)	S1—C18—H18B	109.5
C4—Ru2—Ru3	169.28 (8)	H18A—C18—H18B	109.5
C6—Ru2—Ru3	88.28 (7)	S1—C18—H18C	109.5
C5—Ru2—Ru3	92.72 (7)	H18A—C18—H18C	109.5
C7—Ru2—Ru3	87.07 (7)	H18B—C18—H18C	109.5
C4—Ru2—Ru1	109.84 (8)	C24—C19—C20	118.6 (2)
C6—Ru2—Ru1	147.73 (7)	C24—C19—P1	121.93 (17)
C5—Ru2—Ru1	84.63 (7)	C20—C19—P1	119.49 (17)
C7—Ru2—Ru1	87.89 (7)	C21—C20—C19	121.0 (2)
Ru3—Ru2—Ru1	59.563 (6)	C21—C20—H20	119.5
C9—Ru3—C10	103.41 (10)	C19—C20—H20	119.5
C9—Ru3—C8	89.88 (10)	C22—C21—C20	120.1 (2)
C10—Ru3—C8	93.49 (10)	C22—C21—H21	119.9
C9—Ru3—C11	89.20 (10)	C20—C21—H21	119.9
C10—Ru3—C11	92.35 (10)	C21—C22—C23	119.6 (2)
C8—Ru3—C11	174.14 (10)	C21—C22—H22	120.2
C9—Ru3—Ru1	160.67 (7)	C23—C22—H22	120.2
C10—Ru3—Ru1	95.01 (7)	C22—C23—C24	120.4 (2)
C8—Ru3—Ru1	94.86 (7)	C22—C23—H23	119.8
C11—Ru3—Ru1	84.18 (7)	C24—C23—H23	119.8
C9—Ru3—Ru2	101.48 (7)	C23—C24—C19	120.3 (2)
C10—Ru3—Ru2	154.74 (7)	C23—C24—H24	119.8
C8—Ru3—Ru2	82.25 (7)	C19—C24—H24	119.8
C11—Ru3—Ru2	92.26 (7)	C30—C25—C26	118.7 (2)
Ru1—Ru3—Ru2	60.808 (6)	C30—C25—P1	122.05 (17)
C15—S1—C18	102.86 (11)	C26—C25—P1	119.26 (17)
C12—P1—C19	103.17 (10)	C27—C26—C25	120.7 (2)
C12—P1—C25	102.11 (10)	C27—C26—H26	119.7
C19—P1—C25	102.31 (10)	C25—C26—H26	119.7
C12—P1—Ru1	114.43 (7)	C28—C27—C26	120.1 (2)
C19—P1—Ru1	117.78 (7)	C28—C27—H27	120.0
C25—P1—Ru1	114.99 (7)	C26—C27—H27	120.0
O1—C1—Ru1	172.8 (2)	C29—C28—C27	119.8 (2)
O2—C2—Ru1	177.3 (2)	C29—C28—H28	120.1
O3—C3—Ru1	174.6 (2)	C27—C28—H28	120.1
O4—C4—Ru2	177.3 (2)	C28—C29—C30	120.7 (2)
O5—C5—Ru2	173.0 (2)	C28—C29—H29	119.7
O6—C6—Ru2	178.7 (2)	C30—C29—H29	119.7
O7—C7—Ru2	174.0 (2)	C25—C30—C29	120.1 (2)
O8—C8—Ru3	172.1 (2)	C25—C30—H30	120.0
O9—C9—Ru3	177.3 (2)	C29—C30—H30	120.0
O10—C10—Ru3	177.9 (2)		
			
C19—P1—C12—C17	177.86 (17)	Ru1—P1—C19—C20	−43.3 (2)
C25—P1—C12—C17	71.95 (18)	C24—C19—C20—C21	−0.6 (4)
Ru1—P1—C12—C17	−52.94 (18)	P1—C19—C20—C21	−178.9 (2)
C19—P1—C12—C13	4.2 (2)	C19—C20—C21—C22	0.0 (4)
C25—P1—C12—C13	−101.70 (19)	C20—C21—C22—C23	0.5 (4)
Ru1—P1—C12—C13	133.40 (16)	C21—C22—C23—C24	−0.4 (4)
C17—C12—C13—C14	0.3 (3)	C22—C23—C24—C19	−0.2 (4)
P1—C12—C13—C14	173.99 (17)	C20—C19—C24—C23	0.6 (4)
C12—C13—C14—C15	−1.7 (3)	P1—C19—C24—C23	178.88 (19)
C13—C14—C15—C16	1.5 (3)	C12—P1—C25—C30	6.3 (2)
C13—C14—C15—S1	−178.67 (17)	C19—P1—C25—C30	−100.32 (19)
C18—S1—C15—C14	16.8 (2)	Ru1—P1—C25—C30	130.78 (16)
C18—S1—C15—C16	−163.38 (18)	C12—P1—C25—C26	−174.03 (18)
C14—C15—C16—C17	−0.1 (3)	C19—P1—C25—C26	79.39 (19)
S1—C15—C16—C17	−179.86 (17)	Ru1—P1—C25—C26	−49.5 (2)
C15—C16—C17—C12	−1.3 (3)	C30—C25—C26—C27	−1.5 (3)
C13—C12—C17—C16	1.2 (3)	P1—C25—C26—C27	178.75 (19)
P1—C12—C17—C16	−172.74 (17)	C25—C26—C27—C28	−0.1 (4)
C12—P1—C19—C24	−94.4 (2)	C26—C27—C28—C29	1.3 (4)
C25—P1—C19—C24	11.3 (2)	C27—C28—C29—C30	−1.0 (4)
Ru1—P1—C19—C24	138.48 (17)	C26—C25—C30—C29	1.9 (3)
C12—P1—C19—C20	83.81 (19)	P1—C25—C30—C29	−178.38 (18)
C25—P1—C19—C20	−170.43 (18)	C28—C29—C30—C25	−0.7 (4)

### Hydrogen-bond geometry (Å, º)

**Table:**

*D*—H···*A*	*D*—H	H···*A*	*D*···*A*	*D*—H···*A*
C21—H21···O8^i^	0.95	2.55	3.238 (3)	129

Symmetry code: (i) −*x*, −*y*, −*z*.
